# Inhibition of the different complement pathways has varying impacts on the serum bactericidal activity and opsonophagocytosis against *Haemophilus influenzae* type b

**DOI:** 10.3389/fimmu.2022.1020580

**Published:** 2022-12-12

**Authors:** Lukas Muri, Anna Schubart, Christine Thorburn, Natasa Zamurovic, Thomas Holbro, Michael Kammüller, Gerd Pluschke, Emma Ispasanie

**Affiliations:** ^1^ Molecular Immunology Unit, Swiss Tropical and Public Health Institute, Basel, Switzerland; ^2^ University of Basel, Basel, Switzerland; ^3^ Department Autoimmunity, Transplantation and Inflammation, Novartis Institutes for Biomedical Research, Basel, Switzerland; ^4^ Novartis Pharma AG, London, United Kingdom; ^5^ Translational Medicine-Preclinical Safety, Novartis Institutes for Biomedical Research, Basel, Switzerland; ^6^ Global Drug Development, Novartis Pharma AG, Basel, Switzerland

**Keywords:** *Haemophilus influenzae* type b, serum bactericidal activity (SBA), opsonophagocytosis, complement inhibitors, alternative pathway (AP), vaccination, immunotherapy

## Abstract

Defense against *Haemophilus influenzae* type b (Hib) is dependent on antibodies and complement, which mediate both serum bactericidal activity (SBA) and opsonophagocytosis. Here we evaluated the influence of capsule-specific antibodies and complement inhibitors targeting the central component C3, the alternative pathway (AP; fB, fD), the lectin pathway (LP; MASP-2) and the terminal pathway (C5) on both effector functions. Findings may be relevant for the treatment of certain diseases caused by dysregulation of the complement system, where inhibitors of complement factors C3 or C5 are used. Inhibitors against other complement components are being evaluated as potential alternative treatment options that may carry a reduced risk of infection by encapsulated bacteria. Serum and reconstituted blood of healthy adults were tested for bactericidal activity before and after vaccination with the Hib capsule-conjugate vaccine ActHIB. Most sera had bactericidal activity prior to vaccination, but vaccination significantly enhanced SBA titers. Independently of the vaccination status, both C3 and C5 inhibition abrogated SBA, whereas inhibition of the LP had no effect. AP inhibition had a major inhibitory effect on SBA of pre- vaccination serum, but vaccination mitigated this inhibition for all disease isolates tested. Despite this, SBA-mediated killing of some Hib isolates remained retarded. Even for the most serum-resistant isolate, SBA was the dominating defense mechanism in reconstituted whole blood, as addition of blood cells to the serum did not enhance bacterial killing. Limited Fc receptor-mediated opsonophagocytosis was unmasked when bacterial killing by the membrane attack complex was blocked. In the presence of C3 or C5 inhibitors, addition of post-vaccination, but not of pre-vaccination serum to the blood cells triggered opsonophagocytosis, leading to suppression of bacterial multiplication. Taken together, our data indicate that for host defense against Hib, killing by SBA is more efficient than by blood cell opsonophagocytosis. However, additional defense mechanisms, such as bacterial clearance by spleen and liver, may play an important role in preventing Hib-mediated sepsis, in particular for Hib isolates with increased serum-resistance. Results indicate potentially improved safety profile of AP inhibitors over C3 and C5 inhibitors as alternative therapeutic agents in patients with increased susceptibility to Hib infection.

## Introduction

The encapsulated Gram-negative bacterium *Haemophilus influenzae* is a major cause of invasive bacterial infection in children worldwide (1). There are 6 capsular serotypes (a-f), but in particular isolates from type b (Hib) organisms that possess a polyribosyl ribitol phosphate (PRP) capsule cause invasive disease (2, 3). Although all Hib strains share the same PRP capsule, its expression is variable and dependent on a duplicated 18 kb DNA segment of the Cap b locus (4, 5). Recombination of the two copies can either result in loss of expression or serve as a template for amplification of the capsule gene sequences. A higher copy number leads to an increase in capsule expression and also serum resistance. Infections with Hib can manifest clinically as meningitis, septicaemia, epiglottitis, otitis media and pneumonia (2). However, in the majority of cases, Hib is a commensal of the nasopharynx, and only a minority of carriers suffer invasive disease (6). The highest rate of disease is found in the first 2 years of life, but since the introduction of a PRP- protein conjugate vaccine, invasive Hib disease has virtually been eliminated in countries where Hib vaccines are routinely used with high coverage (1, 7–9). Hib vaccination programs were particularly successful as one single serotype (i.e. type b) caused the majority of infections in a population largely limited to children under the age of 5 (10, 11). Despite the high immunogenicity and efficacy of Hib conjugate vaccines, a few vaccinated children still experience Hib disease. These children may have a defect in immunological priming resulting in lower-avidity antibodies to Hib PRP (2). For Hib, the importance of bactericidal antibodies was demonstrated in 1933, when the presence of bactericidal activity in blood was associated with a reduction of Hib meningitis (12, 13). Using an optimized Hib SBA assay, a strong correlation between anti-PRP IgG concentrations and SBA titers in vaccinated adults and infants was observed (12). Early studies have shown that anti-Hib PRP IgG was not only bactericidal but also opsonic for polymorphonuclear leukocytes in the presence of complement (14, 15). However, the relative importance of different host mechanisms for clearance of invasive Hib disease is not entirely clear.

Data from humans with homozygous C3 deficiencies highlighted the importance of complement during Hib infections, as human C3 deficiency is associated with recurrent and life-threatening bacterial infections by Hib and other encapsulated bacteria (16–18). Hib infections were also associated with deficiencies in C2 (19, 20), C4B (21), factor I (fI) and factor H (fH) (18). The alternative pathway (AP) of complement activation seems to also be involved in Hib immunity as recurrent respiratory Hib infections were observed in monozygous twins with partial factor D deficiency (22). Although patients with C3 complement deficiency rather than individuals with membrane attack complex (MAC, C5b-C9) deficiencies suffer from Hib infections, pointing to C3b opsonization as more important host defense against invasive Hib infection (23, 24), the relative importance of different host mechanisms for clearance of invasive Hib disease has not been studied side-by-side.

Dysregulation of complement activation causes a number of diseases, including paroxysmal nocturnal haemoglobinuria (PNH), C3 glomerulopathy (C3G) and atypical haemolytic uremic syndrome (aHUS) (25). Current treatment of such diseases includes prevention of MAC formation with the mAb eculizumab that binds to C5 and prevents its cleavage to C5a and C5b (25). Recently, the C3 inhibitor pegcetacoplan was also approved for treatment of PNH (26). While MAC formation is involved in uncontrolled lysis of erythrocytes in PNH patients, it is also required for serum bactericidal activity (SBA) and therefore, terminal complement blockage increases the risk of invasive disease by encapsulated bacteria (27–30). *Neisseria meningitidis* infections dominate (31, 32), but it is recommended that children treated with eculizumab also receive vaccinations to prevent *Streptococcus pneumoniae* and Hib infections. As potential alternatives to C3 and C5 inhibitors, AP inhibitors are being developed and we have previously shown that AP inhibition abrogates meningococcal SBA and pneumococcal opsonophagocytosis in vaccine-naïve, but not in vaccinated individuals (33, 34). Here we assessed the impact of complement inhibitors targeting C3, the AP (fB, fD), the lectin pathway (LP) (MASP-2) and the terminal pathway (C5) on both SBA and opsonophagocytic killing of Hib isolates by serum or reconstituted blood from individuals before and after vaccination with a capsule conjugate vaccine.

## Methods

### Ethical approval

All experimental steps involving human specimens were approved by the Ethical Committee of Northwest and Central Switzerland (Ethikkommission Nordwest- und Zentralschweiz (EKNZ), Studie 2018-02341).

### Bacterial isolates and growth conditions

*Haemophilus influenzae* type b case isolates were obtained from the Leibniz Institute. DSMZ-German Collection of Microorganisms and Cell Cultures GmbH (DMS 10001 and DMS 23393) and ATCC (31512 Rab and Eagan mutant ES2593). The Eagan mutant carries a plasmid (ES hktE7::mini–Tn10Cm) allowing selective growth under antibiotic conditions, if required. Bacterial isolates were grown at 37°C with 5% CO_2_ in brain heart infusion broth (BHI, BD) containing 2% Fildes enrichment (Remel) and 2mM cytidine-5′-monophospho-N-acetylneuraminic acid (CMP-NANA, Sigma-Aldrich) to reach logarithmic growth phase (OD_600_ ≈ 0.6).

### Human blood and serum

Six healthy volunteers were recruited upon informed and written consent. Only subject 12 had received an infant Hib vaccination (**Table 1**). All six received one dose of the Hib vaccine (ActHIB^®^, Sanofi Pasteur SA) in the framework of this study. Venous blood was taken before, 2 weeks and 2 months after this vaccination. Vacuette^®^ CAT serum tubes (Greiner Bio-one) were used for collection of sera and preservation of their terminal complement activity was reconfirmed (Complement TCC, Svar Life Science AB). Ethylene diamine tetraacetic acid (EDTA)-anticoagulated whole blood was collected in BD Vacutainer^®^ 4 mL tubes (BD).

**Table 1:** Study subjects and their vaccination status prior to study initiation.

Subject number*	Age	Sex	Previous Hib vaccinations (year)
2	32	F	None
9	33	M	None
11	44	F	None
12	30	M	ProHIBiT^®^ (Berna, 1991, 2 shots)
21	31	F	None
22	45	M	None

*Numbers are not consecutive, as these volunteers were part of a cohort receiving different vaccines. Subjects included in this study are not reflecting a selection, but include all subjects receiving the ActHIB vaccine. Vaccinations and collection of blood were done in March 2020.

### Determination of PRP-specific IgG antibody concentrations

Sera were tested using an ELISA assay. In short, Maxisorp 96-well plates (Nunc) were coated over-night at 4°C with 1 µg/mL Polyribosyl Ribitol Phosphate (PRP, NIBSC 12/306). The human anti-*Haemophilus influenzae* b reference serum (NIBSC 09/222) was used as reference with assigned concentrations of IgG to PRP. After serum incubation for 2 hours at room temperature (RT), plates were incubated for 1 hour at RT with goat anti-human IgG H+L HRP (1:3000, BioRad). Following addition of KPL peroxidase substrate (SeraCare) and 0.5 M sulphuric acid, absorbance was read at 450 nm.

### SBA assay

Serum bactericidal titers were measured as previously described (34), but with modifications. Each reaction mixture contained approximately 400 CFUs of mid-log phase Hib bacteria and 20% human serum with internal complement preserved (35). Bactericidal titers were defined as the interpolated dilution of serum resulting in 50% killing of bacteria after 60 minutes incubation at 37°C compared to the mean CFUs in the control reactions at time 0 (36, 37). Antibody titers were log_10_-transformed using GraphPad Prism v.8.2.1, and concentrations of <5 were assigned the value 2.5 (100% survival). To investigate the contribution of different complement components, the effect of selected complement inhibitors of the alternative, lectin and terminal pathway were analyzed side-by-side. Inhibitors of factor B (iptacopan, LNP023, Novartis (38),), factor D (CMS487, Novartis (38),), C3 (CP-40, Bachem) as well as an anti-C5 antibody (tesidolumab, LFG316, Novartis (39, 40),) and an anti-MASP-2 antibody (produced in-house with the same sequence and properties as narsoplimab) (41, 42) were diluted in Dulbecco’s phosphate-buffered saline (DPBS) and added to the SBA reaction mixture. The activity of the anti-MASP-2 mAb was tested with the Wieslab kit (Svar Life Science AB) for the inhibition of the LP. The IC90 was <10nm. The percent survival after incubation at 37°C for 60 minutes was assessed by plating on chocolate agar PolyViteX (Biomerieux) and compared to CFUs in the control reactions at time 0.

### Killing of Hib in reconstituted whole blood

The reconstituted whole blood opsonophagocytosis assay was performed as previously described with minor modifications (33). Whole blood collected from study volunteers was washed three time with ice-cold DPBS and reconstituted with homologous active serum collected at the above-mentioned time points, and meanwhile preserved at -80°C. Reconstitution with heat-inactivated serum (56°C for 30 minutes at 650 x RPM) served as an internal control. To assess bacterial killing, 80 µL of reconstituted blood and 10 µL of complement inhibitors or DPBS were added to a 96-well plate before inoculating with 10 µL of diluted bacteria at a concentration of 5.32 ± 1.09 x 10^4^ CFU/mL. Intrinsic controls were either serum, heat-inactivated (HI) serum, PBS-washed blood cells, blood cells supplemented with complement-preserved serum, or blood cells supplemented with HI serum. For all these intrinsic controls, 90 µL were added per well to the 96-well plate before adding 10µL of the diluted bacteria. To analyze the effect of blood cells on bacterial killing without serum or plasma, cells were reconstituted with DPBS. After addition of bacteria, the 96-well plates were incubated at 37°C with 5% CO_2_ and bacterial survival was analyzed by repeated sampling of 10 µL after 60, 180 and 300 minutes and plating on chocolate agar PolyViteX (Biomerieux) plates. Agar plates were subsequently incubated overnight at 37°C with 5% CO_2_, colonies were counted manually, and data converted into CFU per mL.

Whole blood assay killing titers were determined as previously described (33). In short, after 4-fold serial dilution of heat-inactivated sera in 96-wells plates, washed blood cells from a vaccine-naïve donor were added before adding diluted bacteria (as described above) and baby rabbit complement (Cedarlane). The 96-well plates were incubated at 37°C with 5% CO_2_ on a rotator. Bacterial killing was analyzed at 3 hours post inoculation. Opsonophagocytic titers represent the reciprocal of the serum dilution with >50% killing compared with bacterial growth in the complement control wells. Serum samples with undetectable titers, representing a titer lower than our lowest dilution (<4), obtained the value 2.

### Statistical analysis

Correlation of SBA and opsonophagocytic titers with the serum anti-PRR IgG concentration was performed using Pearson’s correlation. Correlation analyses included titers assessed pre-vaccination, 2 weeks and 2 months post vaccination for the 6 study participants, except for subject #2, where no 2 week post-vaccination data for opsonophagocytosis are available.

## Results

### Anti-PRP antibody responses and SBA titers elicited by vaccination with ActHIB

**Figure 1:**
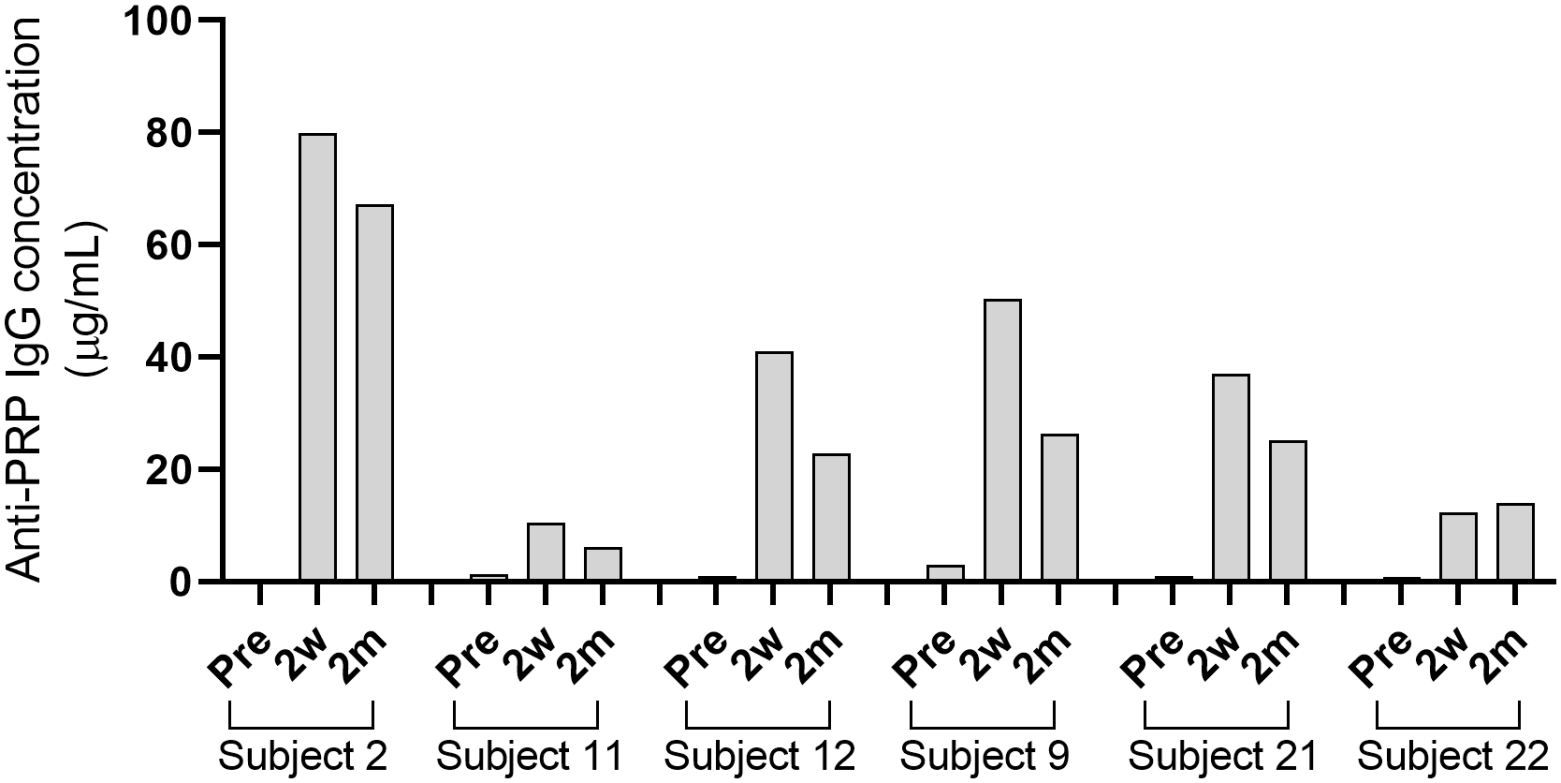
Concentrations of anti-PRP IgG after vaccination with ActHIB. Serum samples were taken before vaccination (Pre), 2 weeks (2w) and 2 months (2m) post-vaccination. Each bar represents the average result of two measurements against the PRP polysaccharide.

Six healthy adult volunteers (**Table 1**) were vaccinated with the Hib capsule conjugate vaccine ActHIB. Development of serum IgG titers against the PRP capsule polysaccharide was determined by ELISA. Pre-vaccination titers ranged from 0.4 to 3.0 µg/mL PRP-specific IgG (**Supplementary Table 1**). For 5 out of 6 subjects, IgG titers peaked at 2 weeks post-vaccination with concentrations between 10.5 to 79.9 µg/mL, and had slightly declined 2 months after vaccination to 6.1 to 67.3 µg/mL (**Figure 1**¸ **Supplementary Table 1**). Only in the case of subject 22, the 2-month titer was moderately higher than the 2-week titer. Titers of subject 12, who had received an infant Hib vaccination, were within the range for all time points. For three subjects, a serum sample 1 year post-vaccination was collected, and anti-PRP IgG concentrations (4.9 to 34.9 µg/mL; **Supplementary Table 1**) were still above the long-term protection threshold, which was estimated by Kayhty et al. (1983) to be 1.0 µg/mL (12, 43).

**Figure 2:**
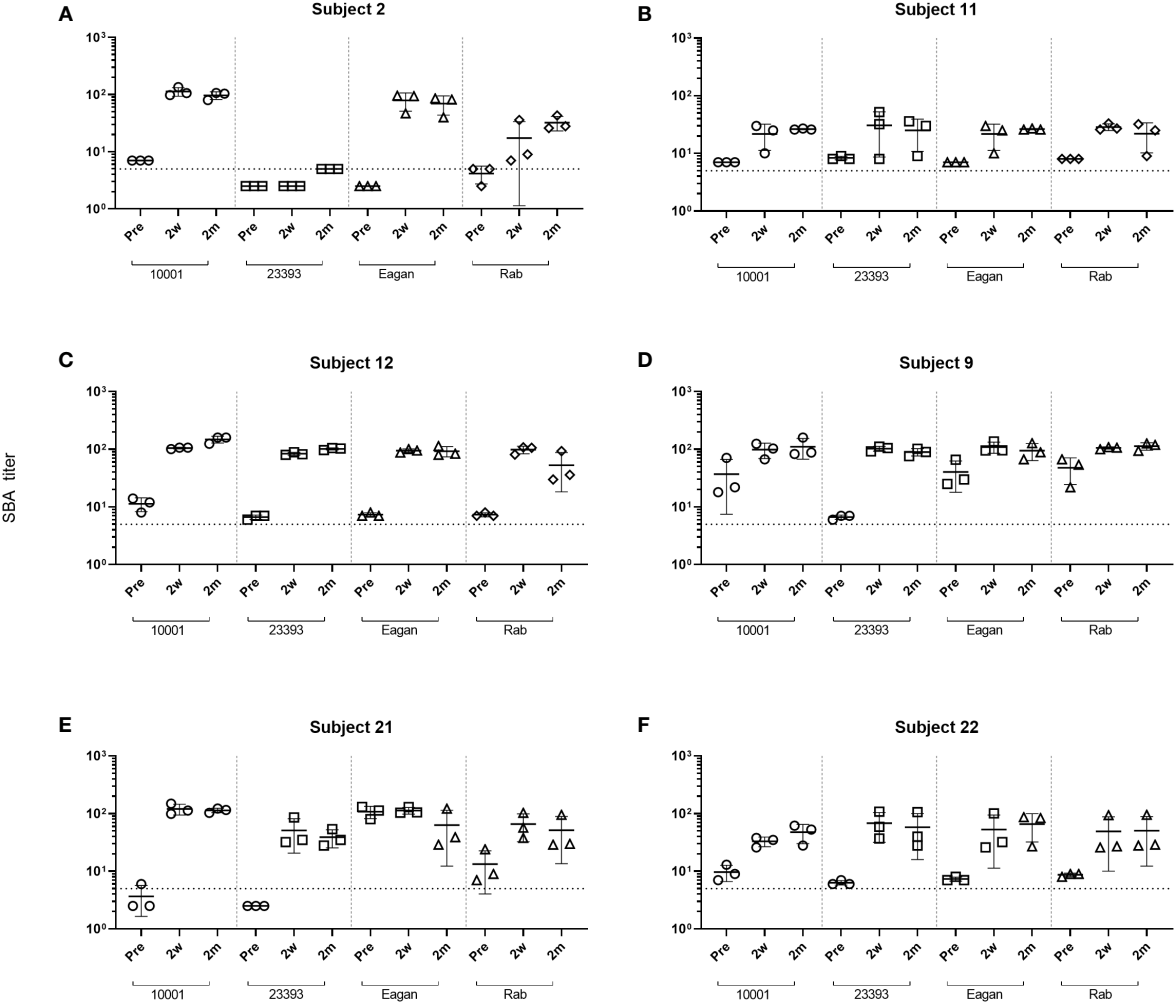
Changes in SBA titers against Hib isolates after ActHIB vaccination. SBA titers of sera taken prior to vaccination (Pre), 2 weeks (2w) and 2 months (2m) post-vaccination from six volunteers **(A–F)**. SBA titers are the reciprocal dilutions of serum that result in 50% killing of the bacteria after 60 minutes incubation. The lowest serum dilution measured was 1:5. Error bars represent the standard deviation of the mean titer of triplicate technical replicates. The bottom dotted line indicates a SBA titer of 5. For serum/isolate combinations that had a SBA titer <5, symbols were set below that line to a SBA titer of 2.5, indicating titers below detection limit.

Following the ActHIB vaccination, serum samples with a broad spectrum of anti-PRP concentrations were obtained to analyze the increase in SBA associated with the vaccine-induced antibody response. The serum samples from all sampling time points had their internal complement preserved and SBA against four Hib case isolates (10001, 23393, Rab and Eagan) was determined. Of the 24 pre-vaccination serum/isolate combinations, 19 had a SBA titer >4 (**Figure 2**). Two weeks after ActHIB vaccination, marked increases in SBA titers were observed for all subjects. SBA titers of sera remained largely stable for 2 months (**Figure 2**). After vaccination, all sera reached a SBA titer of >4 for all Hib isolates at 2 months after ActHIB vaccination. In the three subjects for whom an additional sample was available 1 year after vaccination, SBA titers had slightly decreased to values between 24 and 77, but had remained >4 for all four Hib isolates tested (**Supplementary Table 2**). For unclear reasons, SBA of subject 2 post-vaccination sera (2w and 2m) against isolate 23393 was only slightly enhanced by vaccination, although high anti-PRP IgG titers (>60 µg/mL) were induced by the vaccination (**Figure 1**).

### Effect of complement inhibitors on Hib SBA

**Figure 3:**
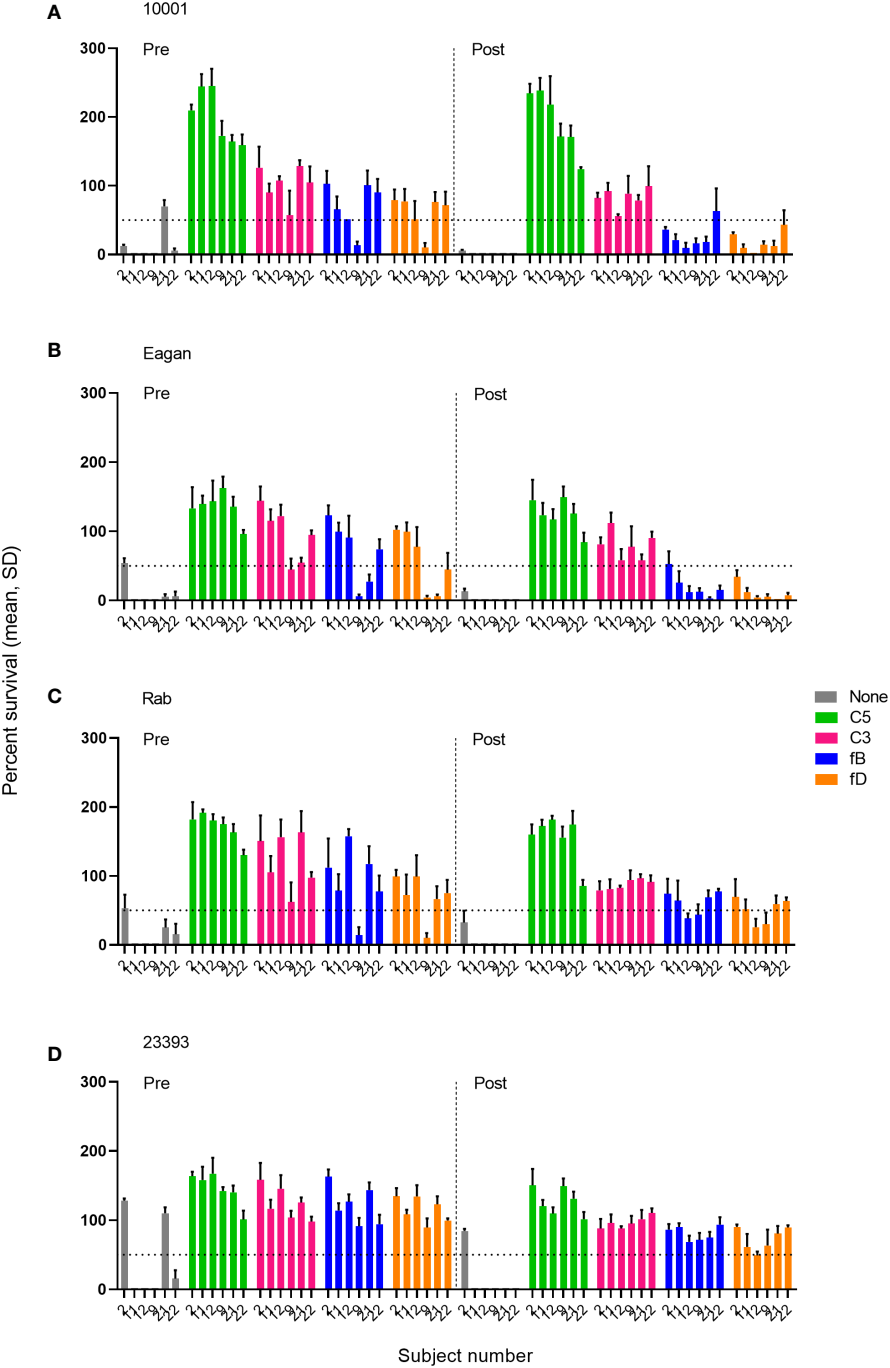
Impact of complement inhibitors targeting the terminal pathway (C5), C3 and the AP (fB, fD) on the survival of Hib isolates **(A–D)** in sera taken prior to vaccination (Pre) or 2 weeks after vaccination with ActHIB (Post). Sera were diluted 1:5 and assayed with the preserved internal complement. Data shown in the graphs are with the highest inhibitor concentration tested (25 µM for the fB inhibitor iptacopan and the fD inhibitor CMS487, 100 µg/mL for the C3 inhibitor CP-40 and the anti-C5 mAb tesidolumab). The horizontal dotted line represents 50% survival of bacteria after 60 minutes incubation, as compared to control wells at time 0. Data for each inhibitor are triplicate technical replicates. SD, standard deviation.

The effect of complement inhibitors on killing of Hib isolates was tested at inhibitor concentrations of 0.05-25 µM for the fB inhibitor iptacopan and the fD inhibitor CMS487 and of 1-100 µg/mL for the C3 inhibitor CP-40, the anti-C5 mAb tesidolumab and the anti-MASP-2 mAb. Typically, full inhibitory activity was already reached at concentrations of 1 µM for the AP inhibitors fB and fD, and of 50 µg/mL for the C3 and C5 inhibitors (**Supplementary Figures 1A–D**). Representative results for C3, the AP (fB and fD) inhibitors and the terminal pathway inhibitor C5 at the highest concentrations tested (25 µM and 100 µg/mL, respectively) are shown in **Figure 3**. As results obtained with serum taken 2 weeks and 2 months after the vaccination were comparable (**Supplementary Figures 1A–D**), **Figure 3** is focused on a comparison of data obtained with sera taken before and 2 weeks after vaccination.

In the absence of complement inhibitors, 79% (19/24) of the pre-vaccination serum/isolate combinations showed substantial SBA with a bacterial survival rate of <50%, compared to the inoculum (**Figure 3**). This SBA may be triggered by natural anti-Hib antibodies in the adult study participants. After immunization this proportion increased further to 96% (23/24). As expected, addition of anti-C5 mAbs abrogated killing of all isolates in both pre- and post- vaccination serum samples. Similarly, the C3 inhibitor blocked killing with 98% (46/48) of the pre- or post-vaccination serum-isolate combinations showing a survival rate of >50%. No SBA inhibition was caused by the anti-MASP-2 mAb inhibiting the LP (**Supplementary Figures 1A–D**).

**Figure 4:**
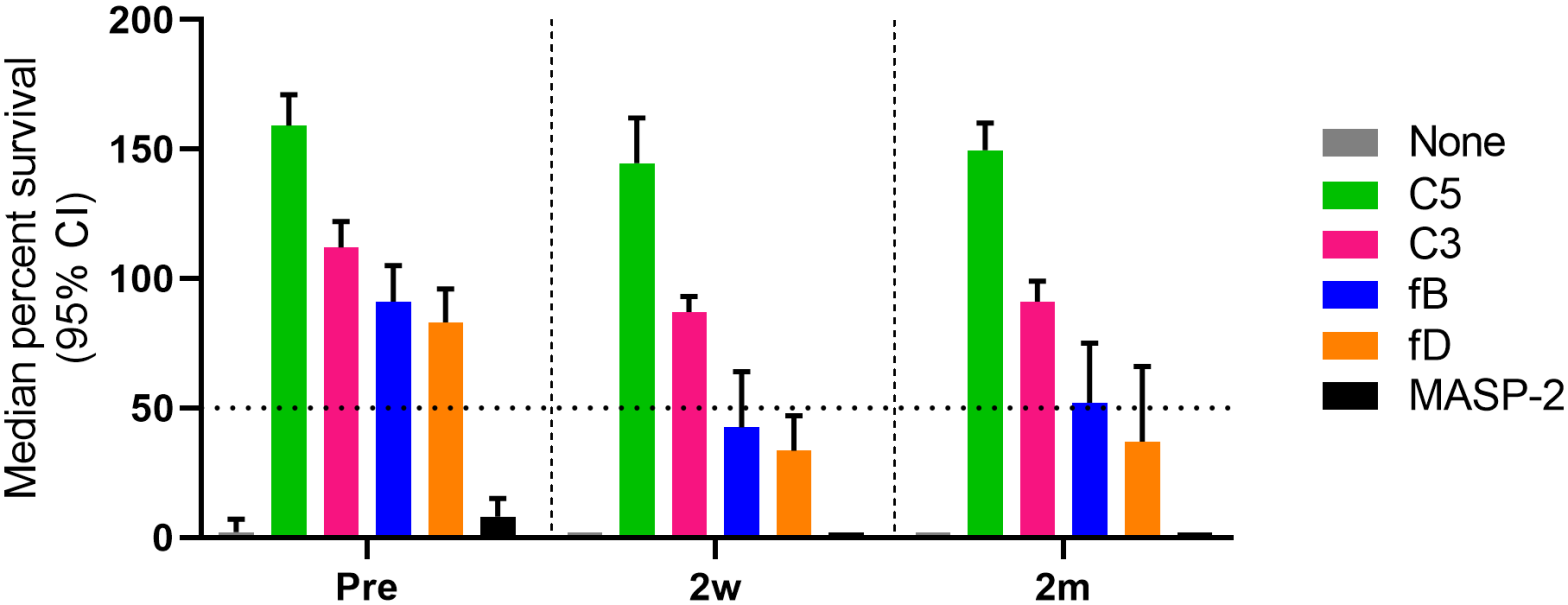
Comparative analysis of the effect of complement inhibitors on the survival of Hib in pre- and post-vaccination sera. Survival data for all subject/Hib isolate combinations were cumulated (three technical replicates for each combination). The horizontal dotted line represents 50% survival of bacteria after 60 min incubation. Shown are results for the highest concentrations of complement inhibitors tested (25 µM for the fB inhibitor iptacopan and the fD inhibitor CMS487, 100 µg/mL for the C3 inhibitor CP-40, the anti-C5 mAb tesidolumab and the anti-MASP-2 mAb). CI, confidence interval.

For AP inhibitors, a marked reduction of SBA was observed with pre-vaccination sera, with only 17% (4/24) of the combinations for fB inhibition and 21% (5/24) for fD inhibition still showing a bacterial survival rate <50% (**Figure 3**). However, this proportion is higher than for the C3 and C5 inhibitors, where a bacterial survival rate <50% was found for only one C3-inhibited and for none of the C5-inhibited combinations. In contrast, AP inhibition had only a minor effect on SBA activity against isolates 10001 and Eagan after vaccination, with 92% (11/12) of the combinations still showing a bacterial survival rate <50% (**Figures 3A, B**). However, against isolates Rab and 23393 (**Figures 3C, D**), AP inhibition reduced SBA markedly and in particular for isolate 23393 a bacterial survival rate <50% was reached with none of the post- vaccination sera. However, immunization reduced bacterial survival in AP-inhibited sera to some degree for all isolates. In spite of the isolate-specific variation, cumulated data for all sera and isolates confirmed that vaccination reduces the effect of the two AP inhibitors on SBA substantially, while C3 and C5 inhibitors abrogate SBA activity independent of the vaccination status of the serum donor (**Figure 4**).

### Killing of Hib by SBA and opsonophagocytosis in reconstituted blood

**Figure 5:**
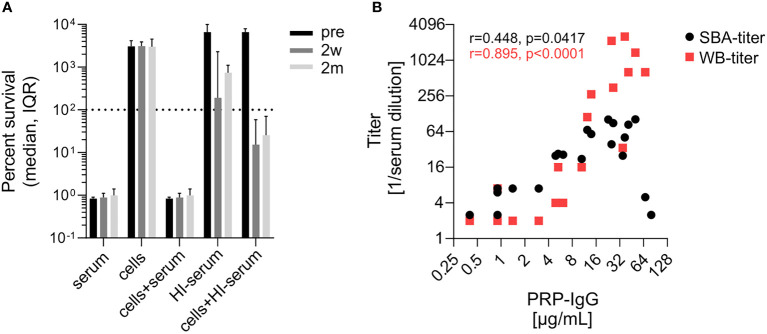
Evidence for complement-independent antibody-mediated opsonophagocytic killing of Hib isolate 23393. **(A)** Killing of Hib isolate 23393 incubated with serum, PBS-washed blood cells, blood cells supplemented with complement-preserved serum, heat-inactivated (HI) serum or blood cells supplemented with HI serum. Cumulated data for all subjects are shown. **(B)** Correlation of bactericidal titers in the SBA and in the reconstituted whole blood assays with the serum anti-PRR IgG concentrations. The Pearson’s correlation analyses include titers assessed pre-vaccination (Pre), 2 weeks (2w) and 2 months (2m) post vaccination. Whole blood killing titers were determined using blood cells reconstituted with serial dilutions of HI human serum and 20% active baby rabbit serum. Titers represent the reciprocal of the serum dilution which yielded >50% killing after 3 hours of incubation. A value of 2 was given to sera with titers below the threshold of 4. WB, reconstituted whole blood assay. Two outlier data points in **(B)** with high anti-PRP IgG titer and low SBA titer correspond to the results obtained with strain 23393 and the two post-vaccination sera (2w and 2m) of subject 2 (see **Figure 2**).

Isolate-specific differences in the sensitivity to SBA prompted us to investigate for the most serum-resistant isolate 23393 to what extent SBA and opsonophagocytic killing complement each other. Killing of isolate 23393 in reconstituted blood was assessed by using cells collected from (EDTA)-anticoagulated blood and supplemented with active homologous pre- or post-vaccination serum with the internal complement preserved. As killing of bacteria by opsonophagocytosis progresses more slowly than SBA-based killing (33), changes in CFU counts were followed up for 5 hours, whereas 1 hour is the standard incubation period for the SBA assays. While three sera showed reduced SBA against isolate 23393, with a bacterial survival rate of >50%, after 1 h in the SBA assays described above (**Figures 2** and **3**), all undiluted pre- and post-vaccination sera showed complete killing after 5 h (**Supplementary Figure 2**). Cumulated data for the reconstituted blood assays showed that killing by active pre- and post- vaccination sera was complete and could not be further enhanced by the addition of blood cells to the serum (**Figure 5A**). In the absence of serum, blood cells on their own had no effect on the multiplication of the bacteria (**Figure 5A**). When the bacteria were incubated with heat-inactivated serum without addition of blood cells, a moderate reduction in the increase of CFUs was observed with post- vaccination sera, but not with pre-vaccination serum. This may be related to some aggregation of the bacteria by the capsule-specific antibodies present in post-vaccination sera with heat-inactivated complement. However, addition of blood cells to the bacteria suspended in heat-inactivated post-vaccination serum led to a marked decrease in bacterial survival, indicative of opsonophagocytic killing triggered by the interaction of Fc-receptors with the capsule-specific antibodies bound to the bacterial cell surface. In contrast, multiplication of bacteria was undisturbed when the blood cells were reconstituted with heat-inactivated pre-vaccination sera lacking capsule-specific antibodies. Bactericidal titers in the SBA and in the reconstituted whole blood assay both correlated with the anti-PRP IgG titers (**Figure 5B**).

**Figure 6:**
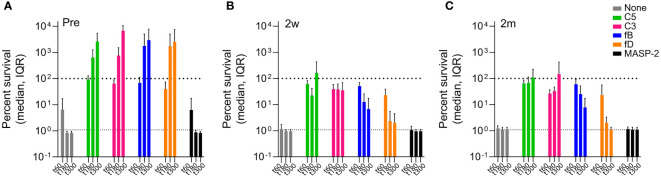
Effect of complement inhibitors on killing of Hib isolate 23393 in reconstituted whole blood before and after ActHIB vaccination. Cumulated data for all study subjects are shown. The upper dashed lines corresponds to the amount of bacteria inoculated at time 0 (100% survival), while the lower dashed line represents the lower detection limit (< 100 CFU/mL) for bacterial quantification. Bacterial killing was assessed using PBS-washed EDTA-anticoagulated blood cells reconstituted with undiluted active serum, comparing serum taken before **(A)**, 2 weeks **(B)** and 2 months **(C)** after ActHIB vaccination. CFUs were quantified 1, 3 and 5 hours after inoculation, represented by the 3 bars. The following inhibitor concentration were analyzed: fB inhibitor (iptacopan): 25 µM; fD inhibitor (CMS487): 25 µM; C3 inhibitor (CP-40): 50 µg/mL; anti-C5 mAb (tesidolumab): 50 µg/mL; and anti-MASP-2 mAb: 50 µg/mL.

After 5h of incubation in reconstituted blood, consisting of blood cells supplemented with either pre- or post-vaccination serum, isolate 23393 was efficiently killed by the combined action of SBA and opsonphagocytic killing (**Figure 6**, **Supplementary Figure 3**). However, in the presence of C3, C5, fB or fD inhibitors, massive multiplication of the bacteria was observed when cells were supplemented with pre-vaccination serum, whereas inhibition of the lectin pathway with MASP-2-specific mAbs had no effect (**Figure 6A**). In contrast, C3 and C5 inhibition still abrogated efficient killing of bacteria in post-vaccination sera, but no marked increase in CFU was observed (**Figures 6B, C**). In the absence of MAC-mediated SBA, antibody-mediated opsonophagocytosis thus appears to be efficient enough to contain bacterial multiplication.

In comparison to C3 and C5 inhibition, the effect of AP (fB and fD) inhibition was much more limited when blood cells were supplemented with post-vaccination sera. After 5h of incubation, CFU counts were close to or below the limit of detection, demonstrating that even in the absence of the AP-mediated amplification loop of the classical pathway (CP), the bacteria are efficiently killed by the combined action of SBA and opsonophagocytosis.

## Discussion

After clinical introduction of inhibitors that block all three complement pathways for the treatment of complement-related diseases, novel approaches are targeting individual pathways to mitigate safety concerns. However, also for therapeutics targeting individual complement pathways, the potentially reduced safety concerns have to be addressed, and in particular the risk of invasive bacterial infection associated with the treatment is yet to be evaluated. Several genetic complement deficiencies have been identified through a high risk of infection with a limited spectrum of mostly encapsulated bacteria. Individuals with deficiencies in terminal complement components and in patients treated with the C5-specific inhibitor such as eculizumab carry increased susceptibility to invasive meningococcal infections (28) Increased susceptibility to Hib infection has been observed in individuals in particular with early complement component (C1, C2, C3, C4) deficiencies, but not C5 deficiency (19, 44–46) indicating that host defense against invasive Hib infection does not entirely rely on the lytic activity of the MAC of complement. It has been suggested that C3b opsonization contributes to host defense (23, 24) as patients with C3 complement deficiency suffer from Hib infections rather than individuals with membrane attack complex (MAC, C5b-C9) deficiencies. Here, we have studied *in vitro* the relative importance of SBA and opsonization-induced phagocytosis by blood cells side-by-side *in vitro*.

Literature suggests that anti-PRP antibodies can trigger killing of Hib both through SBA and opsonization-induced phagocytosis by human polymorphonuclear leukocytes (47–49). The standard approach for the evaluation of immune responses to Hib conjugate vaccination is the determination of anti-PRP capsular antibody concentrations by ELISA. Proposed short- and long-term protective thresholds are ≥0.15 and ≥1.0 µg/mL (43), respectively. However, functionality of the anti-PRP antibodies may not always correlate directly with antibody titers. While Townsend et al., 2014 (12) suggested that an anti-PRP level of 1.0 µg/mL corresponds to an SBA titer of 8, they also identified children with high anti-PRP IgG titer, but low SBA, and vice versa. Similarly, we found one example, subject 2, in which SBA against one particular isolate (23393) remained low despite high anti-PRP IgG titers. Under our assay conditions, the anti-PRP IgG concentrations in the sera of the adult study subjects before vaccination were between 0.37-3.0 µg/mL. Bactericidal activity, which may be triggered by natural antibodies specific for both the capsule and for other cell surface antigens, was found for most serum/isolate combinations. SBA titers of the pre-vaccination sera ranged from 2.5 (no killing) to 108 (**Figure 2**, **Supplementary Table 2**). AP inhibitors reduced this killing activity both in the SBA and in the reconstituted whole blood assay, indicating that the AP-mediated amplification loop of the CP plays a key role when antibody titers are limiting. After vaccination, anti-PRP IgG concentrations of 6-80 µg/mL were reached and with a single exception, the SBA titers against the four isolates tested increased substantially. AP inhibition did not block killing of the bacteria when these high concentrations of anti-capsule antibodies were present. Overall, SBA titers correlated with the anti-PRP IgG concentrations. While C3 and C5 inhibition blocked SBA of both pre- and post-vaccination sera, inhibition of the LP had no effect on SBA.

Besides SBA, opsonophagocytosis has been identified as alternative defense mechanism after immunization with Hib conjugate vaccines (14, 15, 47, 50). These studies used purified polymorphonuclear neutrophils supplemented with serum. Here we used whole blood cells to analyze the contribution of opsonophagocytosis to the killing of the most serum-resistant isolate 23393 in reconstituted blood. In the absence of complement inhibitors, killing of isolate 23393 by SBA was retarded, with incomplete killing after the standard 1 hour incubation in the standard SBA assay, but complete killing in the reconstituted blood assay was seen after 5 hours. In reconstituted blood, the effect of SBA dominated and opsonophagocytosis was only unmasked when bactericidal activity was inhibited by the addition of C3 or C5 inhibitors. While in the absence of SBA, massive bacterial multiplication was observed when blood cells were supplemented with pre-vaccination sera, multiplication was contained by Fc-receptor-mediated opsonophagocytosis when post-vaccination sera were used. These data indicate that opsonophagocytosis by blood cells can contribute to bacterial clearance after immunization, but that this contribution is limited. In particular for more serum-resistant Hib isolates, additional mechanisms, such as clearance by the reticuloendothelial system of the spleen or by hepatic neutrophil extracellular traps and macrophages may supplement SBA and play an important role in preventing Hib-mediated sepsis (51). As clearance of encapsulated bacteria by these mechanisms is already operational in infants (52) spleen and liver may play a key role in protecting children in the first 2 years of life, when susceptibility to invasive Hib infection is high in unvaccinated infants (53).

We have previously shown that AP inhibitors, which are being developed as potential alternatives to therapeutic C3 and C5 inhibitors, abrogate the killing of meningococci by SBA in pre-vaccination serum and the killing of pneumococci by opsonophagocytosis in pre-vaccination blood (33, 34). In contrast, in serum or blood from vaccinated individuals, AP inhibitors had only a limited effect on bacterial killing. In the present study, C3 and C5 inhibition blocked killing of Hib by SBA both in pre- and post-vaccination serum completely, while LP inhibition had no effect. Reduction of SBA by the fB and fD inhibitors in pre-vaccination sera was less pronounced than for the C3 and C5 inhibitors and immunization reduced the inhibitory effect of the AP inhibitors for all Hib isolates tested.

Related to ethical considerations, one limitation of the present investigation was the use of sera from adults rather than from children under the age of five, which are most affected by Hib infection. While complement components are already synthesized early in fetal life, the complement activity in newborns seems to be lower than in adults (54). It has been reported that the C1q and properdin levels are low and fB levels are high in children between 1-5 years (55) and that the concentration of complement proteins C3 and C4 is constant in children under 18 years (56). The reported generally reduced complement activity may contribute to the higher susceptibility of newborns and infants to invasive infection by various encapsulated bacteria. To our knowledge, no specific data are available on the age related development of SBA and opsonophagocytic activity against Hib. However, for opsonophagocytic activity against meningococci, a peak has been observed in the 6- to 12- month age group (57).

In summary, our results highlight the significance of MAC-mediated SBA for Hib clearance and indicate that clearance by blood cells has limited efficacy pre-vaccination. While C3 and C5 inhibition permit Hib multiplication in both pre- and post- vaccination blood, AP inhibitors interfere much less with Hib killing by SBA. From the perspective of infection risks caused by encapsulated bacteria, *in vitro* results with Hib, meningococci and pneumococci indicate potential advantages of AP inhibitors over C3 and C5 inhibitors, in particular, when treatment is combined with prior vaccination of patients (33, 34). Clinical data are required to substantiate that AP inhibitors are possible alternative therapeutic agents with improved safety profile.

## Data availability statement

The original contributions presented in the study are included in the article/**Supplementary Material**. Further inquiries can be directed to the corresponding author.

## Ethics statement

The studies involving human participants were reviewed and approved by Ethical Committee of Northwest and Central Switzerland (Ethikkommission Nordwest- und Zentralschweiz (EKNZ), Studie 2018-02341). The patients/participants provided their written informed consent to participate in this study. Written informed consent was obtained from the individual(s) for the publication of any potentially identifiable images or data included in this article.

## Author contributions

LM and EI contributed to study conceptualization, data acquisition, analysis and interpretation and drafted the manuscript. GP contributed to study conceptualization and design, data analysis and interpretation and revised the manuscript. AS, CT, NZ, TH and MK contributed to study conceptualization and design and revised the manuscript. All authors contributed to the article and approved the submitted version.
