# Supplementary material for: Inhibition of the different complement pathways has varying impacts on the serum bactericidal activity and opsonophagocytosis against Haemophilus influenzae type b
Source: Front Immunol. 2022 Dec 12;13:1020580. doi: 10.3389/fimmu.2022.1020580 (PMC9791579; doi:10.3389/fimmu.2022.1020580)
Supplement: Supplementary file 1 [file DataSheet_1.docx]

Supplementary Material

# Supplementary Data

| **Subject** | 2 | | | | 11 | | | | 12 | | | | 9 | | | 21 | | | 22 | | |
| --- | --- | --- | --- | --- | --- | --- | --- | --- | --- | --- | --- | --- | --- | --- | --- | --- | --- | --- | --- | --- | --- |
| **Serum sample** | Pre | 2w | 2m | yr | Pre | 2w | 2m | yr | Pre | 2w | 2m | yr | Pre | 2w | 2m | Pre | 2w | 2m | Pre | 2w | 2m |
| PRP specific IgG (µg/mL) | 0.4 | 79.9 | 67.3 | 34.9 | 1.4 | 10.5 | 6.1 | 4.9 | 0.9 | 41.0 | 22.8 | 5.3 | 3.0 | 50.4 | 26.4 | 0.9 | 37.0 | 25.1 | 0.9 | 12.4 | 13.9 |

|  | **Subject** | 2 | | | | 11 | | | | 12 | | | | 9 | | | 21 | | | 22 | | |
| --- | --- | --- | --- | --- | --- | --- | --- | --- | --- | --- | --- | --- | --- | --- | --- | --- | --- | --- | --- | --- | --- | --- |
|  | **Serum sample** | Pre | 2w | 2m | yr | Pre | 2w | 2m | yr | Pre | 2w | 2m | yr | Pre | 2w | 2m | Pre | 2w | 2m | Pre | 2w | 2m |
| Mean SBA titer | 10001 | 7 | 113 | 97 | 34 | 8 | 31 | 25 | 28 | 11 | 105 | 148 | 56 | 37 | 98 | 110 | 4 | 120 | 114 | 10 | 33 | 48 |
|  | Eagan | 2.5 | 79 | 69 | 44 | 7 | 25 | 23 | 36 | 7 | 95 | 93 | 77 | 40 | 109 | 95 | 108 | 113 | 63 | 7 | 53 | 66 |
|  | Rab | 4 | 17 | 32 | 26 | 8 | 29 | 22 | 24 | 7 | 98 | 53 | 27 | 48 | 105 | 113 | 13 | 66 | 51 | 9 | 49 | 51 |
|  | 23393 | 2.5 | 2.5 | 5 | 25 | 7 | 22 | 26 | 25 | 7 | 84 | 102 | 27 | 7 | 103 | 89 | 2.5 | 51 | 39 | 6 | 68 | 58 |

**Supplementary Table 1. Summary of the PRP-specific IgG concentrations in human serum samples from subjects vaccinated with the conjugate vaccine ActHIB.** The concentration of PRP polysaccharide specific IgG in the serum samples were determined using the human anti-Hib reference serum from NIBSC (09/222) with known concentration of IgG, IgM and IgA towards the PRP polysaccharide. Abbreviations: pre vaccination serum (Pre); serum taken 2 weeks (2w), 2 months (2m), 1 year (yr) after vaccination with ActHIB.

**Supplementary Table 2. Summary of the average SBA titers of the human serum samples of subjects vaccinated with the polysaccharide conjugate vaccine ActHIB.** SBA titers are the reciprocal dilutions of serum that result in 50% killing of the bacteria after 60 minutes incubation. A lack of SBA is indicated with the value 2.5. The lowest serum dilution tested was 1:5. Abbreviations: pre vaccination serum (Pre); serum taken 2 weeks (2w), 2 months (2m), 1 year (yr) after vaccination with ActHIB.

**
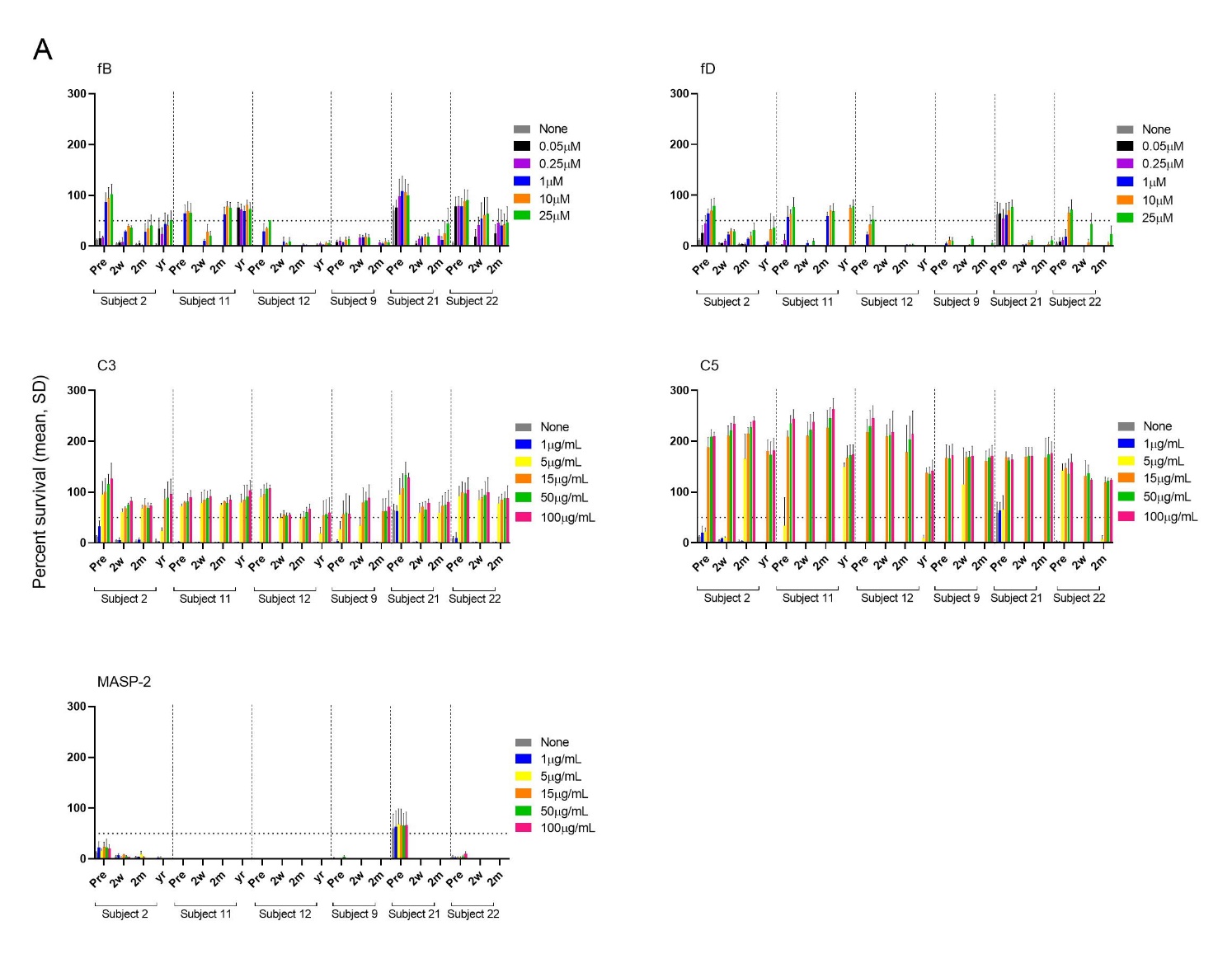
**

**
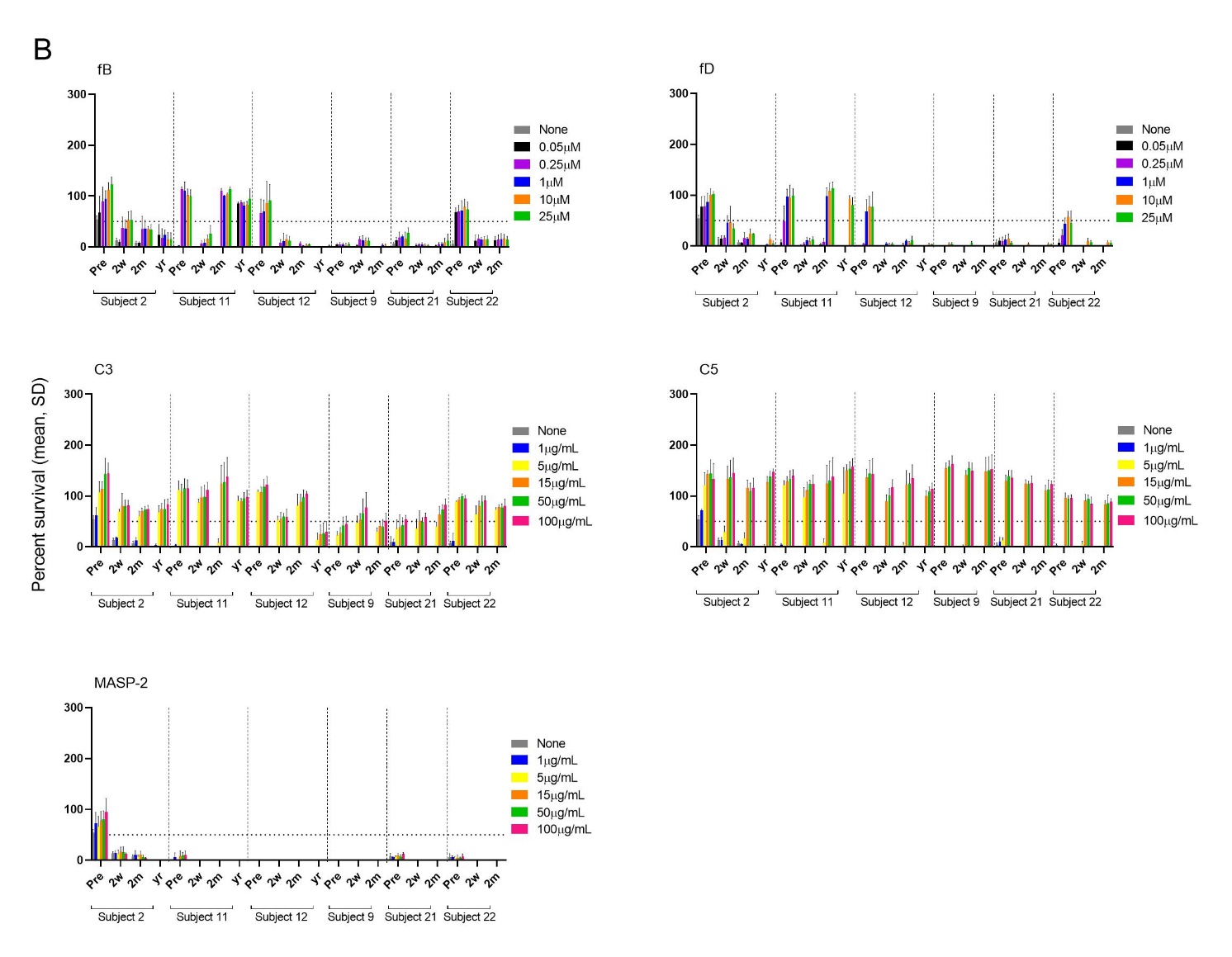
**

**
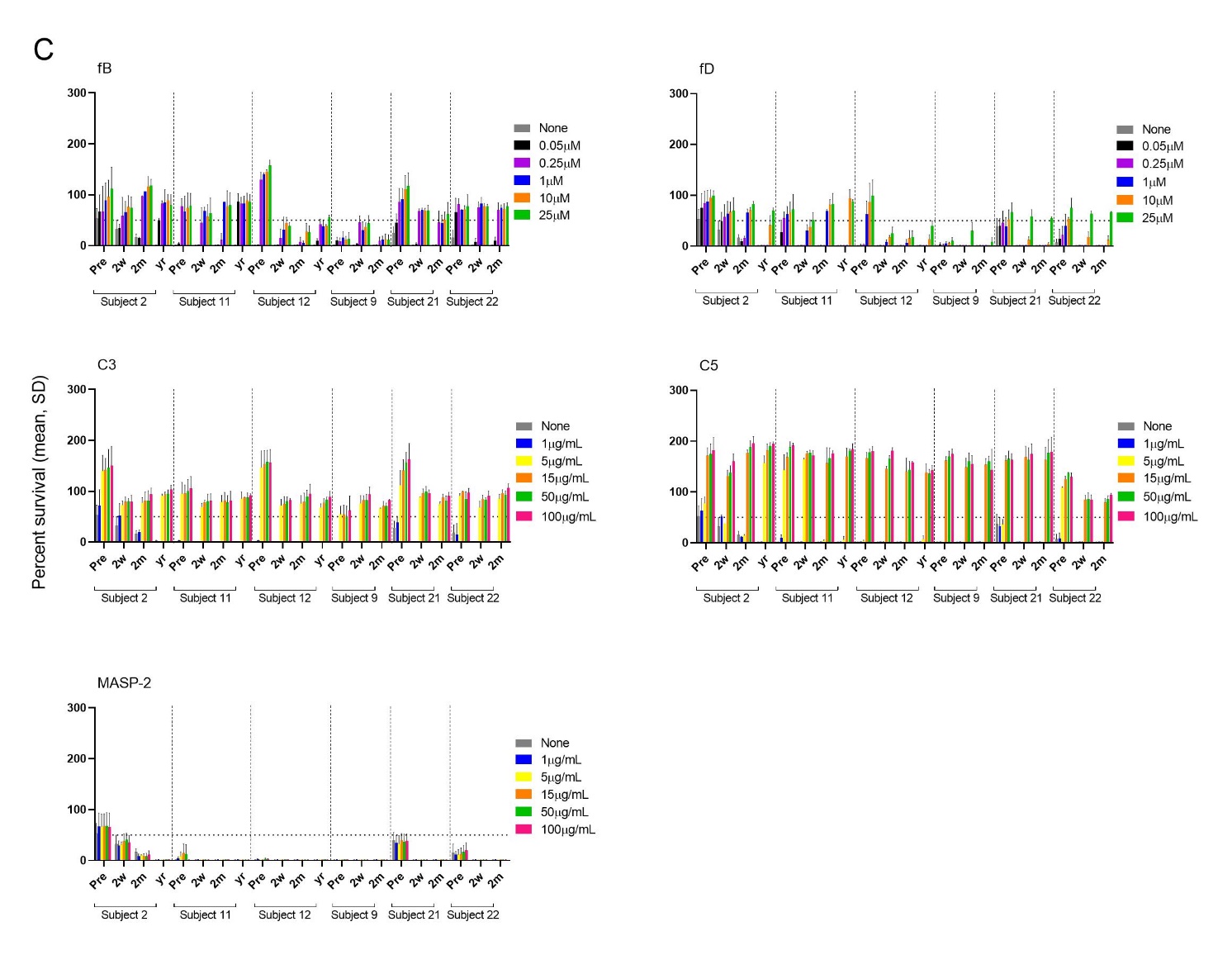
**

**
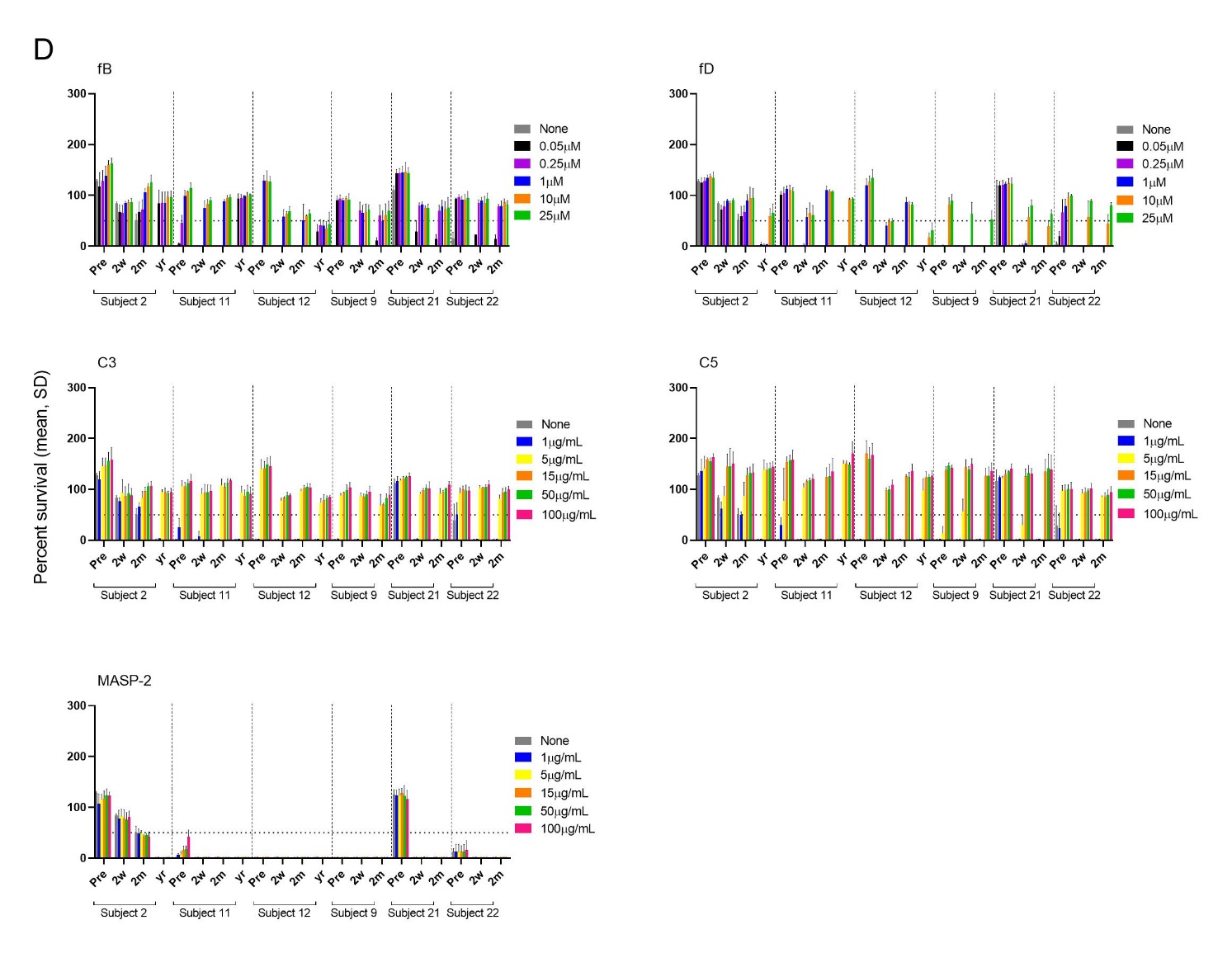
**

**Supplementary Figure 1. The effect of complement inhibitors on the survival of the Hib isolates in pre- and post- ActHIB vaccination sera.** All subjects were vaccinated with the conjugate PRP polysaccharide vaccine ActHIB. All sera samples for each subject were diluted 1:5 and assayed with the internal complement and against Hib case isolates 10001 (**A**), Eagan (**B**), Rab (**C**) and 23393 (**D**). The horizontal dotted line represents 50% killing of bacteria after 60 minutes incubation with different concentrations of factor B (fB) inhibitor iptacopan , factor D (fD) inhibitor CMS487 , C3 inhibitor (C3) CP-40 , anti-C5 monoclonal antibody (mAb; C5) tesidolumab and anti-MASP-2 mAb (MASP-2) narsoplimab . For each graph, grey bars are test sera without added inhibitors. Data for each inhibitor are triplicate technical replicates. SD: standard deviation of mean. Abbreviations: pre vaccination serum sample (Pre); 2 weeks (2w), 2 months (2m), 1 year (yr) after vaccination with ActHIB.


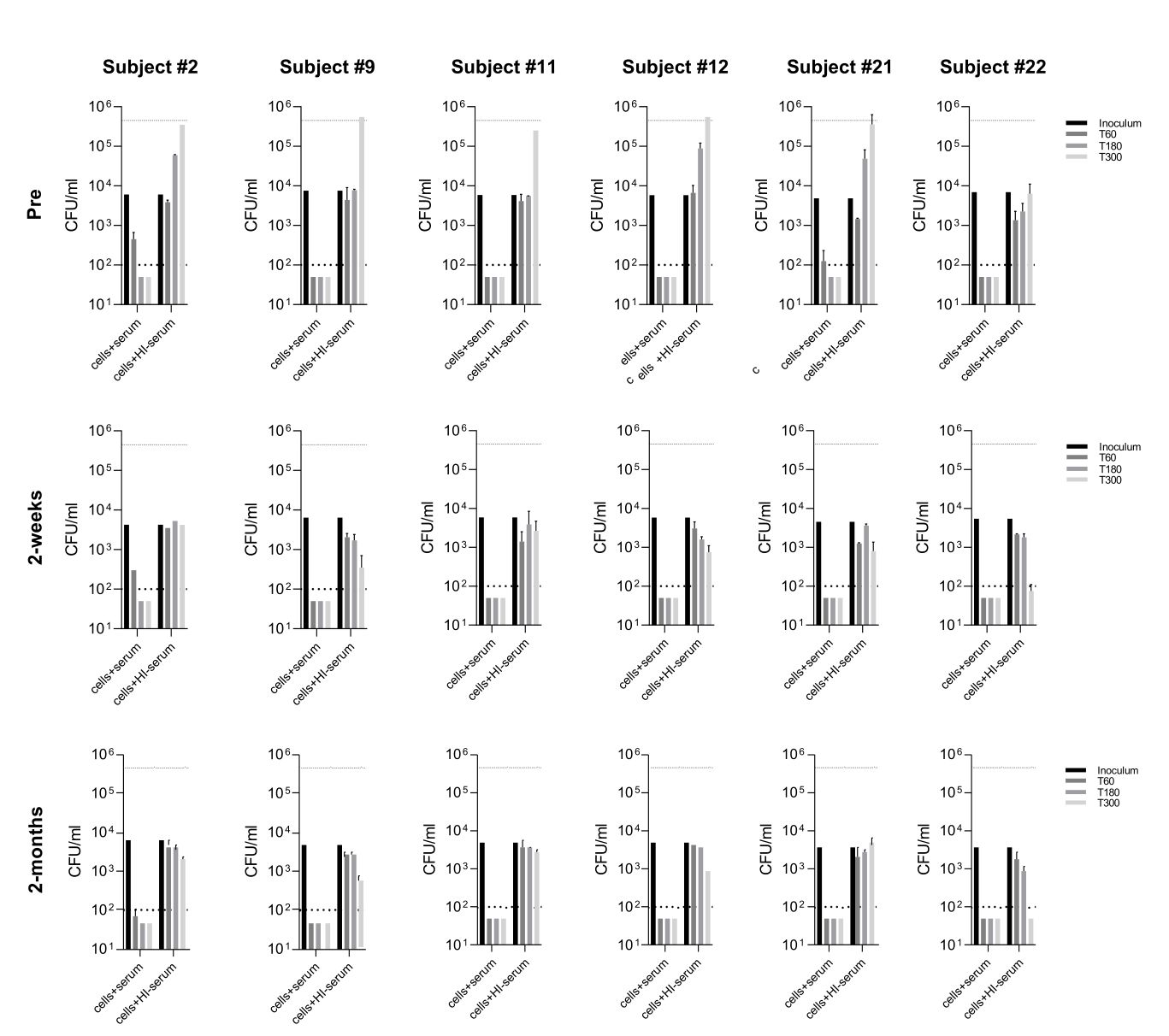


**Supplementary Figure 2. Killing of *H. influenzae* isolate 23393 incubated with PBS-washed blood cells supplemented with complement-preserved or heat-inactivated (HI) serum.** Individual data from all six assessed subjects are shown. Bacterial killing was assessed using PBS-washed EDTA-anticoagulated blood cells reconstituted with undiluted active or HI serum, comparing serum taken before, 2 weeks and 2 months after ActHIB vaccination. CFUs were quantified 1, 3 and 5 hours after inoculation, represented by the 4 bars, with the black bar representing the amount of inoculated bacteria.

**
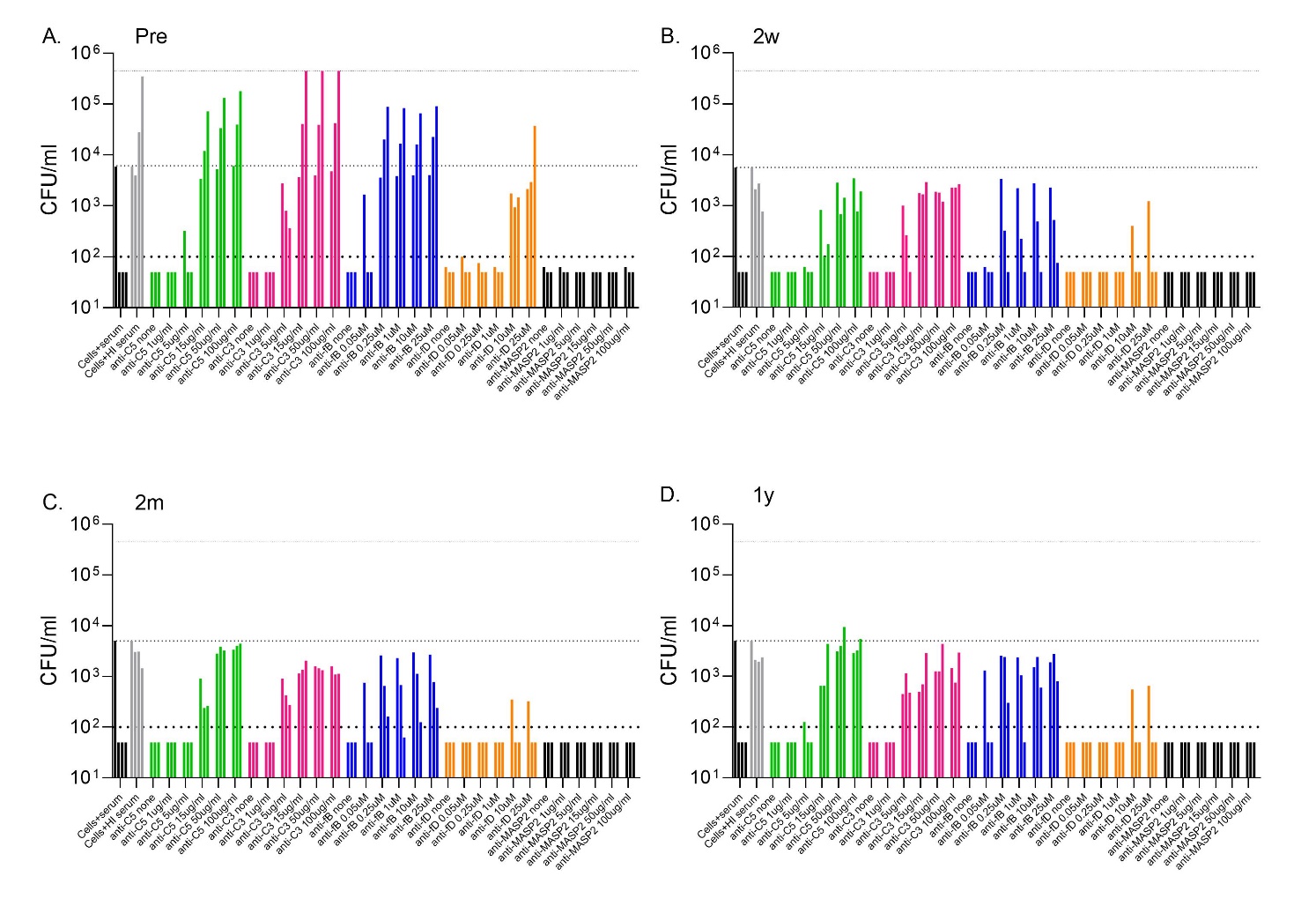
Supplementary Figure 3. Effect of complement inhibitors on killing of Hib strain 23393 in reconstituted whole blood before and after ActHIB vaccination.** Cumulated data for all study subjects are shown. The upper dashed lines corresponds to the amount of bacteria inoculated at time 0, while the lower dashed line represents the lower detection limit (< 100 CFU/mL) for bacterial quantification. Bacterial killing was assessed using PBS-washed EDTA-anticoagulated blood cells reconstituted with undiluted active serum, comparing serum taken before (A), 2 weeks (B) 2 months (C) and 1 year (D) after ActHIB vaccination. CFUs were quantified 1, 3 and 5 hours after inoculation, represented by the 3 bars comparing the following complement inhibitors: fB inhibitor (iptacopan); fD inhibitor (CMS487); C3 inhibitor (CP-40); anti-C5 mAb (tesidolumab) and anti-MASP-2 mAb (narsoplimab). 1 year data was only available for three subjects (#2, #11, #12).

**
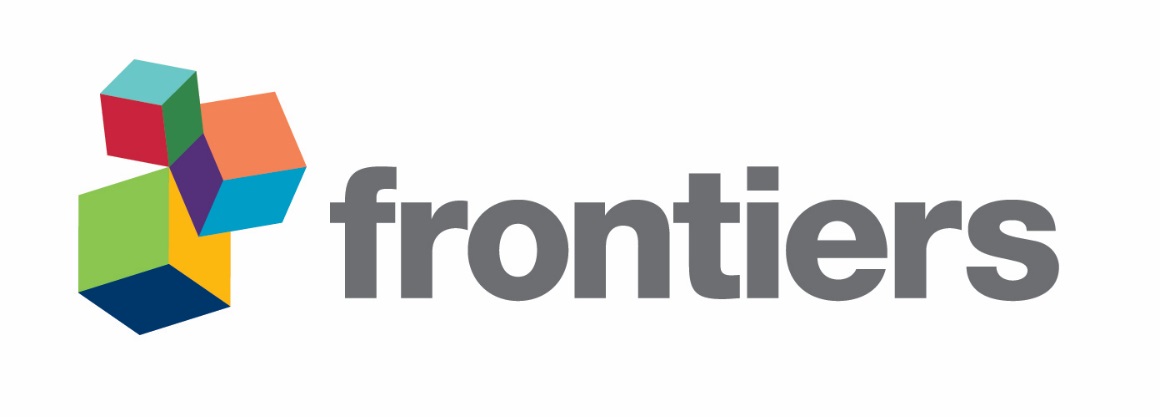
**
